# Supplementary material for: Multistage and transmission-blocking targeted antimalarials discovered from the open-source MMV Pandemic Response Box
Source: Nat Commun. 2021 Jan 11;12:269. doi: 10.1038/s41467-020-20629-8 (PMC7801607; doi:10.1038/s41467-020-20629-8)
Supplement: Supplementary file 1 — Supplementary Information [file 41467_2020_20629_MOESM1_ESM.pdf]

SUPPLEMENTARY INFORMATION

Multistage and transmission-blocking targeted antimalarials discovered from the open-source MMV Pandemic Response Box

Janette Reader[1a], Mariëtte E. van der Watt[1a], Dale Taylor[2], Claire Le Manach[2], Nimisha Mittal[3], Sabine Ottilie[3], Anjo Theron[4], Phanankosi Moyo[1], Erica Erlank[5], Luisa Nardini[5], Nelius Venter[5], Sonja Lauterbach[6], Belinda Bezuidenhout[6], Andre Horatscheck[2], Ashleigh van Heerden[1], Natalie J. Spillman[7], Anne N. Cowell[3], Jessica Connacher[1], Daniel Opperman[1], Lindsey M. Orchard[8], Manuel Llinás[8, 9], Eva S. Istvan[7], Daniel E. Goldberg[7], Grant A. Boyle[2], David Calvo[10], Dalu Mancama[4], Theresa L. Coetzer[6], Elizabeth A. Winzeler[3], James Duffy[11], Lizette L. Koekemoer[5], Gregory Basarab[2], Kelly Chibale[2,12], Lyn-Marié Birkholtz[1]*

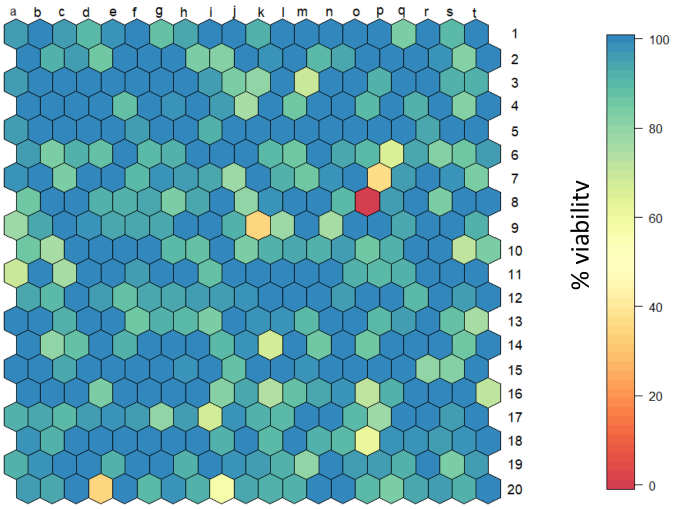

Supplementary Fig. 1: Supra-hexoplot for % viability of CHO cells after treatment with compounds at 2 uM. Figure supplementary to Figure 2A. Compound identities, placement and individual values are provided in supplementary File S1.

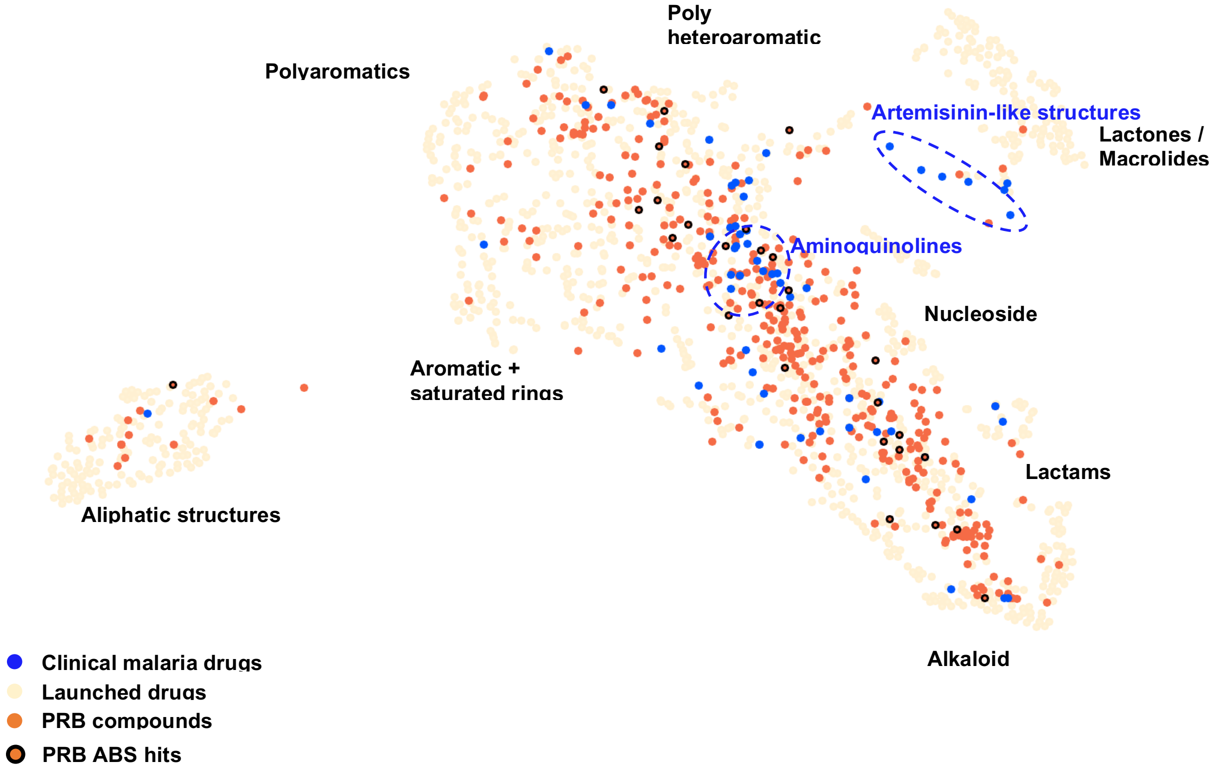

Supplementary Fig. 2: Structural diversity of the MMV PRB compared to Malaria Clinical Drugs. MMV PRB (red dots) and Malaria Clinical Drugs (blue dots) chemical spaces are plotted in the Launched Drugs Space (beige dots). The Launched Drug Space is available as standard dataset within the StarDrop V 6.6 software. Supplementary figure to Figure 4.

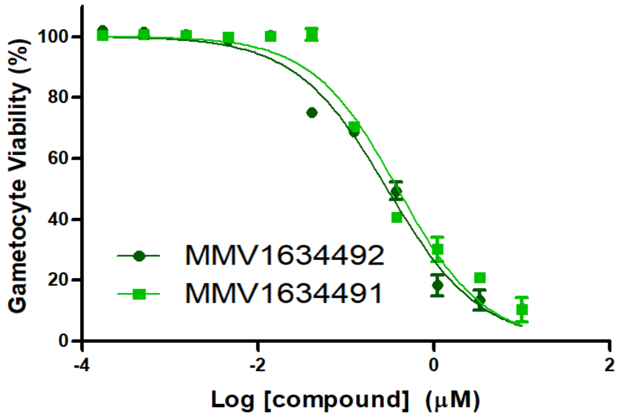

Supplementary Fig. 3: Gametocyte-specific compounds were enriched for azole antifungals, including MMV1634491 and MMV1634492, with dose-responses of these two compounds indicated after 48 h exposure to stage IV/V gametocytes and evaluated on the PrestoBlue® assay. Data are from three independent biological repeats (n=3), each performed in technical triplicates, mean ± S.E. Supplementary figure to Fig 5.

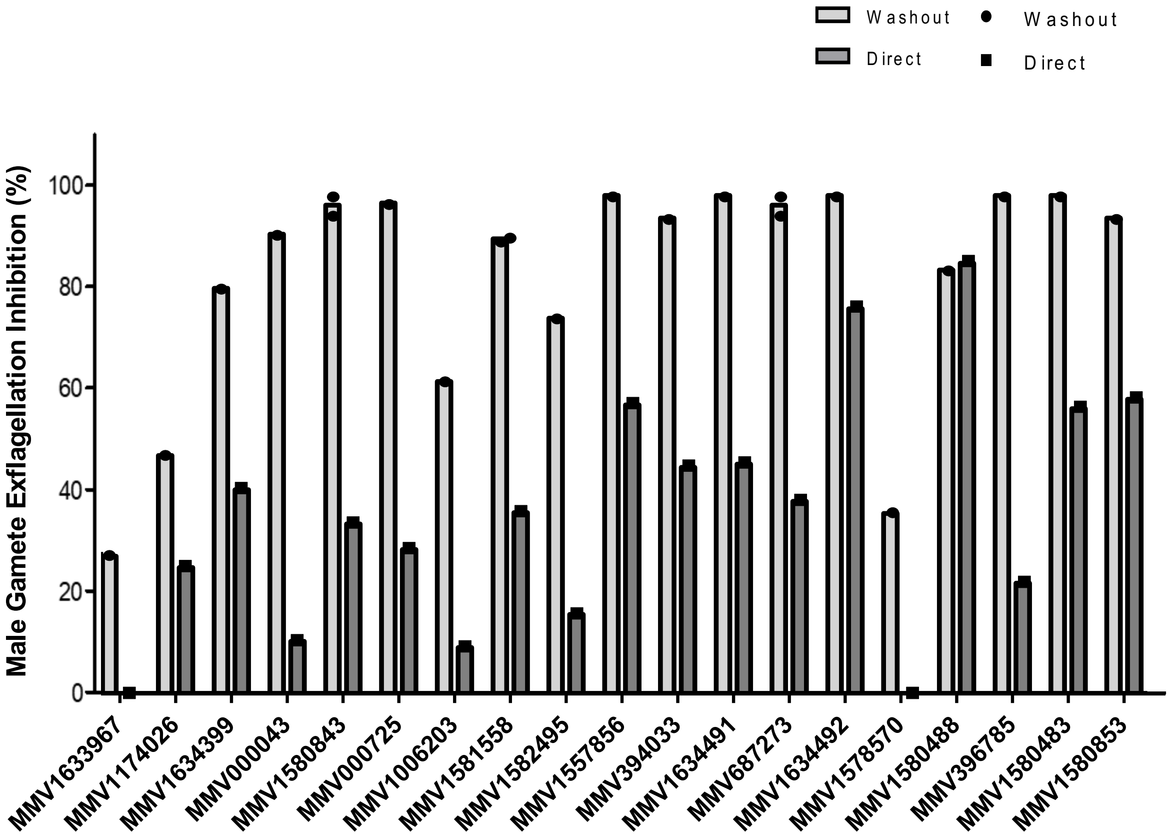

Supplementary Fig. 4: Inhibition of male gamete exflagellation in both direct or washout format. Figure supplementary to Figure 5B. Direct: compound added during induction of male exflagellation (30 min incubation), washout: stage V gametocytes treated with compound for 48 h, compound washed out and male gamete exflagellation tested in absence of compounds. Data are from duplicate experiments ± S.D.

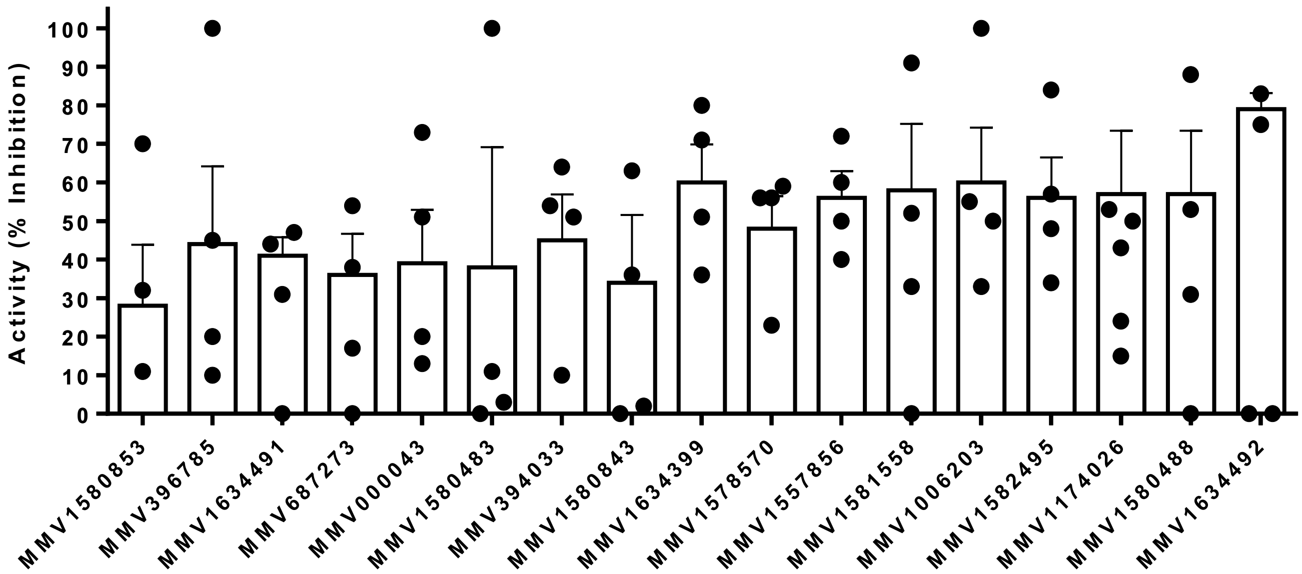

Supplementary Fig. 5: Transmission-blocking activity (%TBA) on SMFA. Figure supplementary to Figure 5C. Data are from n=4 biological repeats, mean ± S.E. indicated (except for MMV1580853, where only n=3 was performed).

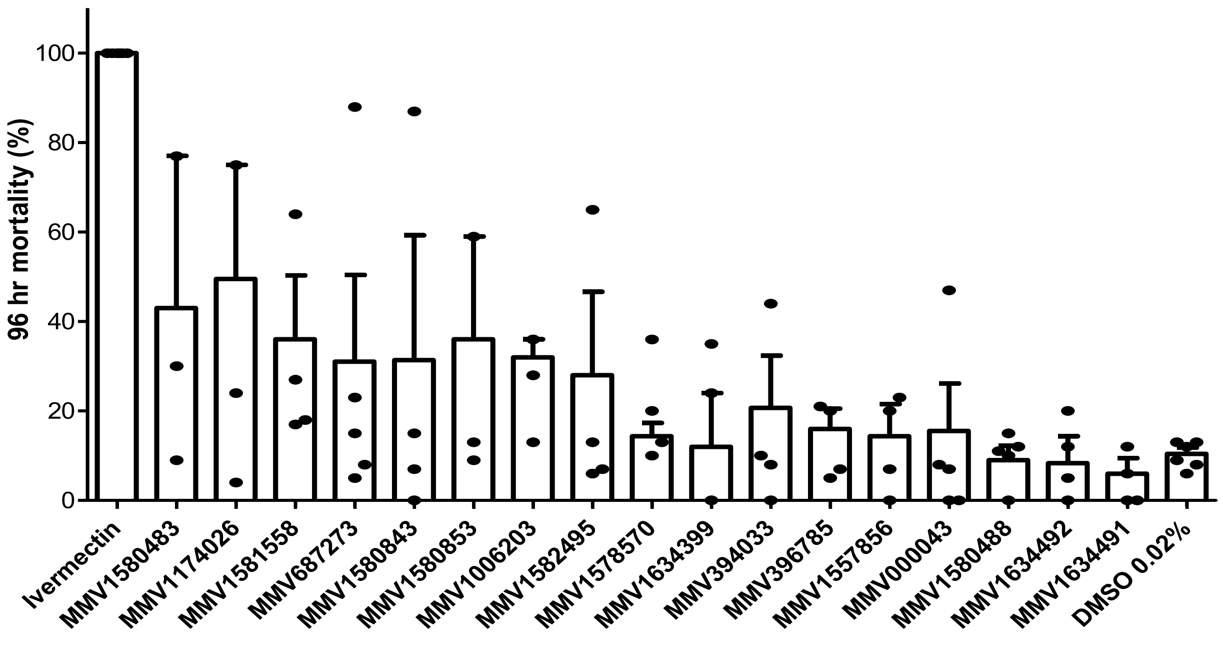

Supplementary Fig. 6: Endectocidal activity of 17 compounds with gametocytocidal activity. Compounds were screened at 2 μM and mortality of *An. coluzzii* (G3) determined after 4 days, compared to Ivermectin (2 μM) as drug control, with DMSO (0.02%) as vehicle control. Data are from n=4 independent biological repeats (except for MMV1580488, MMV000043, MMV687237which is n=5 and MMV1634399, MMV1006203, MMV.1580483 and MMV1174205 which is n=3), mean ± S.E.

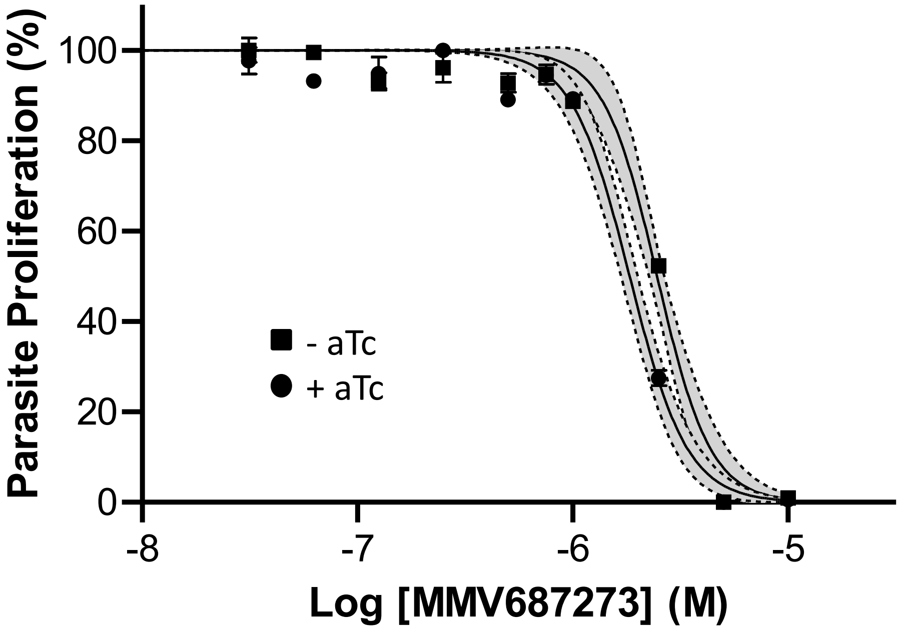

Supplementary Fig. 7: Dose-response of MMV687273 (SQ109, 72 h pressure) against a *Pf*NCR1-aptamer inducible knockdown line, where *Pf*NCR1 expression is controlled by anhydrotetracycline (aTc), with wild type levels of PfNCR1 retained in presence of aTc (+aTc, 500 nM, 1.8 ± 0.08 µM), and *Pf*NCR1 knock-down induced due to aTc removal (-aTc, 2.43 ± 0.15 µM) (unpaired t-test *p*=0.0024). Data are from FACS analysis of parasitemia of acridine orange stained parasites, in triplicates (n=3), mean ± S.E. 95% confidence intervals on each point indicated as ribbons. Supplementary figure to Figure 7.

```
        10         20         30         40         50
MTKVAVEKEE PGVVYKVAGS LVIAENMSGT RMYELAKVGW NKLVGEIIRL

        60         70         80         90        100
EGNYAYIQVY EDTSGLSVGD PVIKTGNALS VELGPGILDN IYDGIQRPLE

       110        120        130        140        150
RIANVCGDVY IYKGIDMTSL DHDKQWQFYA DKKLKLNDIV TGGDIFGFVD

       160        170        180        190        200
ENKLFKEHKI MAPPNAKGRL TYIAPDGSYT LKDKIFELEY QGKKYTYGLS

       210        220        230        240        250
HLWPVRDPRP VLEKVTGDTL LLTGQRVLDS LFPTVQGGTC AIPGAFGCGK

       260        270        280        290        300
TCVSQALSKY SNSEVIIYVG CGERGNEMAE ILSDFPELTT KVDNEDVGIM

       310        320        330        340        350
QRTCLVANTS NMPVAAREAS IYTGITLCEY FRDMGYNATM MADSTSRWAE

       360        370        380        390        400
ALREISGRLA EMPADSGYPA YLGARLASFY ERAGKVKCIG SPSRIGSITI

       410        420        430        440        450
VGAVSPPGGD FSDPVTTATM SIVQAFWGLD KKLAQRKHFP SVNWSTSFSK

       460        470        480        490        500
YVRQLEQYFD NFDQDFLSLR QKISDILQQE SDLNDIVQLV GKDSLSEDQK

       510        520        530        540        550
VVMEVAKIIR EDFLQQNAFS DYDYMCPLQK TVGMMRIICH FYAQCLRTLQ

       560        570        580        590        600
EYDSRERKIG WGSIYNTLRP TINKITHMKF ENPKNSDEYF KKYFKALEEE

       610
ITVGLRNLME K
```

Nucleotide binding domain
Mutation sites Q225, R353, bold, yellow
Walker A motif (P-loop)
Non-homologous regulatory region

Supplementary Fig. 8: Sequence of the *P. falciparum* V-type H^+-ATPase subunit A (PF3D7_1311900) with domains indicated in coloured bars underneath the sequence as per InterPro analysis https://www.ebi.ac.uk/interpro/protein/UniProt/Q76NM6/. The two mutation sites Q225K and R353K are indicated. Supplementary figure to Fig 7.

Supplementary table 1: Assay reproducibility for each assay platform (as described in the methods section) as evaluated by Z'-factor parameter according to the equation:
Z' = 1-[3(SD positive + SD background)/Mean positive – Mean background)]. ABS = asexual blood stages, GC = gametocyte.

Assay platform	Z'-factor
PfNf54 ABS pLDH colorimetric assay	0.63
PfDd2 ABS SYBR Green I fluorescence	0.51
PfNF54 GC ATP bioluminescence	0.864
PfNF54 GC luciferase reporter assay	0.656
PfNF54 GC PrestoBlue fluorescence	0.904
Pb liver assay	0.44
CHO MTT colorimetric	0.64
HepG2 Cell-Titer Glo luminescence	0.52

Supplementary table 2: 95% confidence intervals (CI) for IC_{50} values of the pan-reactive compounds Birinapant and AZD-0156. Supplementary data to Figure 3.

	**MMV1557856** (IC_{50}, µM, ± S.E.)	**CI**	**MMV1580483** (IC_{50}, µM, ± S.E.)	**CI**
**ABS *Pf*NF54**	0.986 ± 0.055	0.8032-1.177	3.998 ± 0.799	2.667-4.161
**ABS *Pf*Dd2**	0.236 ± 0.00	0.17-0.32	0.776 ± 0.079	0.56-1.06
***Pf* stg IV/V gc**	0.135 ± 0.001	0.1149-0.1749	0.236 ± 0.001	0.1983-0.277
***Pb* liver stage**	0.128 ± 0.011	0.0334-0.431	1.055 ± 0.035	0.584-5.47

Supplementary table 3: Single nucleotide polymorphisms (SNPs) identified in SQ109-resistant clones compared to the SQ109-sensitive 3D7 parent clone (270 ± 83 nM). SNPs were found in the V-type H+-ATPase subunit A (*Pf*3D7_1311900) in all four clones, and in the AAA family ATPase (*Pf*3D7_0711000) for only two clones. Quality refers to the phred-scaled quality score for the alternate base call.

Gene	Chr	SQ109-resistant clone	IC_{50} (nM ± S.E.)	Position	Quality	Ref	Alt	Amino acid change
***Pf*3D7_1311900** (V-type H+ATPase subunit A)	13	SQ1	637 ± 95	508957	6100.46	G	A	R353K
		SQ2	686 ± 123					
		SQ3	632 ± 41	508572	4232.46	C	A	Q225K
		SQ4	572 ± 37					
***Pf*3D7_0711000** (AAA family ATPase)	7	SQ3	632 ± 41	488912	1458.46	G	T	D687N
		SQ4	572 ± 37					
