# Supplementary material for: Multistage and transmission-blocking targeted antimalarials discovered from the open-source MMV Pandemic Response Box
Source: Nat Commun. 2021 Jan 11;12:269. doi: 10.1038/s41467-020-20629-8 (PMC7801607; doi:10.1038/s41467-020-20629-8)
Supplement: Supplementary file 3 — Description of Additional Supplementary Files [file 41467_2020_20629_MOESM3_ESM.pdf]

Description of Additional Supplementary Files

Supplementary Data 1: Data of the supra-hexagonal plot in Figure 2A

Supplementary Data 2: Complete dataset of all MMV PRB compounds' activity on Plasmodium life cycle stages

Supplementary Data 3: Full SMFA dataset to support Figure 5C

Supplementary Data 4: Transcriptome analysis of MMV1580488 (ML324) treated parasites to support Figure 6C.
