# Supplementary material for: Multistage and transmission-blocking targeted antimalarials discovered from the open-source MMV Pandemic Response Box
Source: Nat Commun. 2021 Jan 11;12:269. doi: 10.1038/s41467-020-20629-8 (PMC7801607; doi:10.1038/s41467-020-20629-8)
Supplement: Supplementary file 8 — Reporting Summary [file 41467_2020_20629_MOESM8_ESM.pdf]

nature research

Corresponding author(s): Lyn-Marie Birkholtz

Last updated by author(s): 17 Nov 2020

Reporting Summary

Nature Research wishes to improve the reproducibility of the work that we publish. This form provides structure for consistency and transparency in reporting. For further information on Nature Research policies, see our Editorial Policies and the Editorial Policy Checklist.

Statistics

For all statistical analyses, confirm that the following items are present in the figure legend, table legend, main text, or Methods section.

n/a	Confirmed	
☐	☒	The exact sample size (*n*) for each experimental group/condition, given as a discrete number and unit of measurement
☐	☒	A statement on whether measurements were taken from distinct samples or whether the same sample was measured repeatedly
☐	☒	The statistical test(s) used AND whether they are one- or two-sided<br>*Only common tests should be described solely by name; describe more complex techniques in the Methods section.*
☒	☐	A description of all covariates tested
☒	☐	A description of any assumptions or corrections, such as tests of normality and adjustment for multiple comparisons
☐	☒	A full description of the statistical parameters including central tendency (e.g. means) or other basic estimates (e.g. regression coefficient) AND variation (e.g. standard deviation) or associated estimates of uncertainty (e.g. confidence intervals)
☐	☒	For null hypothesis testing, the test statistic (e.g. *F*, *t*, *r*) with confidence intervals, effect sizes, degrees of freedom and *P* value noted<br>*Give P values as exact values whenever suitable.*
☒	☐	For Bayesian analysis, information on the choice of priors and Markov chain Monte Carlo settings
☐	☒	For hierarchical and complex designs, identification of the appropriate level for tests and full reporting of outcomes
☐	☒	Estimates of effect sizes (e.g. Cohen's *d*, Pearson's *r*), indicating how they were calculated

Our web collection on statistics for biologists contains articles on many of the points above.

Software and code

Policy information about availability of computer code

Data collection	Drug classes and biological pathways or protein targets were identified for each compound after text and structure searches of PubChem (https://pubchem.ncbi.nlm.nih.gov/), DrugBank (https://www.drugbank.ca/) and SciFinder (https://sso.cas.org). Reference genome data was obtained from PlasmoDB (vs 13, www.plasmodb.org.)
Data analysis	Chemical space analysis was performed with StarDrop v 6.6 (https://www.optibrium.com/stardrop/) based on structure similarities. The launched drug space was generated from the data file available with Stardrop software. The antimalarial drug space was generated using marketed antimalarial drugs and compounds undergoing clinical trials. The connectivity network was constructed by clustering the compounds using the FragFP descriptor (Tanimoto similarity index >0.50) in OSIRIS DataWarrior v 5.0.0 (www.openmolecules.org). The network was visualized using Cytoscape v 3.7.2. Supra-hexagonal maps was generated in Rstudio 1.1.456 with the RColorBrewer R package. IC50 values were determined in CDD vault (https://www.collaborativedrug.com/) or Graphpad Prism v6 or 8.3.0 that was also used for statistical analyses and to generate graphs were appropriate. Agilent Feature Extractor Software (v 11.5.1.1) was used to analyse Agilent microarray slides with the limma and marray packages in R (v3.2.3, www.r-project.org) for normalisation. Protein structure analysis was performed with UniProt: https://www.uniprot.org/uniprot/Q76NM6, and InterPro: https://www.ebi.ac.uk/interpro/protein/UniProt/Q76NM6/. Structure fitting and visualisation was performed with UCSF Chimera (https://www.cgl.ucsf.edu/chimera/). Dotblot image quantification was performed with Image J v 1.53a.

For manuscripts utilizing custom algorithms or software that are central to the research but not yet described in published literature, software must be made available to editors and reviewers. We strongly encourage code deposition in a community repository (e.g. GitHub). See the Nature Research guidelines for submitting code & software for further information.

Data

Policy information about availability of data

All manuscripts must include a data availability statement. This statement should provide the following information, where applicable:

- Accession codes, unique identifiers, or web links for publicly available datasets
- A list of figures that have associated raw data
- A description of any restrictions on data availability

All data presented in this study are provided in the article and supplementary material or available from the corresponding author upon request. Transcriptome data is available from GEO (accession code GSE157420), whole genome sequencing data from NCBI Sequence Read Archive (PRJNA659232) and structural data from Swiss Model Repository (Q76NM6). Code used to analyze data are available at GitHub (https://github.com/Ash-bot/Beehive-inhibition-plots).

Field-specific reporting

Please select the one below that is the best fit for your research. If you are not sure, read the appropriate sections before making your selection.

- [x] Life sciences
- [ ] Behavioural & social sciences
- [ ] Ecological, evolutionary & environmental sciences

For a reference copy of the document with all sections, see nature.com/documents/nr-reporting-summary-flat.pdf

Life sciences study design

All studies must disclose on these points even when the disclosure is negative.

Sample size	NA, chemistry was limited to number of compounds in box. All other samples were associated with parasitaemia used in the different assays performed, and each assay was performed in at least 3 biological independent experiments.
Data exclusions	No data was excluded
Replication	All experiments performed in at least technical duplicates for with biological replication throughout, in the majority of instances with at least three independent biological repeats (with technical triplicates in each biological repeat). All replicates were successful.
Randomization	Not applicable for the study design which was dependent on a standardised compound assay cascade
Blinding	No data was blinded in any of the assays performed, identities of the compounds assayed was available from the start.

Reporting for specific materials, systems and methods

We require information from authors about some types of materials, experimental systems and methods used in many studies. Here, indicate whether each material, system or method listed is relevant to your study. If you are not sure if a list item applies to your research, read the appropriate section before selecting a response.

Materials & experimental systems

n/a	Involved in the study	
[ ]	[x]	Antibodies
[ ]	[x]	Eukaryotic cell lines
[x]	[ ]	Palaeontology and archaeology
[ ]	[x]	Animals and other organisms
[x]	[ ]	Human research participants
[x]	[ ]	Clinical data
[x]	[ ]	Dual use research of concern

Methods

n/a	Involved in the study	
[x]	[ ]	ChIP-seq
[x]	[ ]	Flow cytometry
[x]	[ ]	MRI-based neuroimaging

Antibodies

Antibodies used	Anti-H3K9me3 antibody (Abcam ab8898), Anti-H3K9ac (Abcam ab4441), anti-H3 core (Abcam ab1791) and goat anti-rabbit horseradish peroxidase (HRP)-conjugated secondary antibody (Abcam ab6721) .
Validation	The primary antibodies Anti-H3K9me3 antibody (Abcam ab8898), Anti-H3K9ac (Abcam ab4441), anti-H3 core (Abcam ab1791) were all validated for use against Plasmodium falciparum, with citations: Bunnik EM, et al. Changes in genome organization of parasite-specific gene families during the Plasmodium transmission stages. Nat Commun 9, 1910 (2018); Coetzee N, et al. Quantitative chromatin proteomics reveals a dynamic histone post-translational modification landscape that defines asexual and sexual Plasmodium falciparum parasites. Sci Rep 7, 607 (2017).

Eukaryotic cell lines

Policy information about cell lines

Cell line source(s)	HepG2 and CHO cells were sourced from ATCC
Authentication	HepG2 and CHO cells Lines were initially authenticated by ATCC.
Mycoplasma contamination	The cell lines used in the study was regularly tested for Mycoplasma contamination and was free of mycoplasma contamination at the time of the study.
Commonly misidentified lines (See ICLAC register)	No commonly misidentified cell lines were used in this study.

Animals and other organisms

Policy information about studies involving animals; ARRIVE guidelines recommended for reporting animal research

Laboratory animals	Mosquito species: Anopheles coluzzii. Colony: G3. Age used 2-7 day old females were used for feeding and dissected when females were 13-15 days old.
Wild animals	No wild animals was used in this study
Field-collected samples	No field collected samples were used in this study
Ethics oversight	This work holds ethical approval from the University of Pretoria Health Sciences Ethics Committee (506/2018); University of Cape Town: AEC017/026; University of the Witwatersrand Human Research Ethics Committee (M130569) and Animal Ethics Committee (20190701-7O); CSIR Research Ethics Committee (Ref 10/2011) and Scripps Research's Normal Blood Donor Service (NBDS), with approval under IRB Number 125933.

Note that full information on the approval of the study protocol must also be provided in the manuscript.
